# Application of Regulatory Cell Death in Cancer: Based on Targeted Therapy and Immunotherapy

**DOI:** 10.3389/fimmu.2022.837293

**Published:** 2022-03-10

**Authors:** Xiaochen Qi, Quanlin Li, Xiangyu Che, Qifei Wang, Guangzhen Wu

**Affiliations:** First Affiliated Hospital, Dalian Medical University, Dalian, China

**Keywords:** regulatory cell death (RCD), immunotherapy, tumor microenvironment, caspase, GSDM, PARP, ECM, DAMPs

## Abstract

The development of cancer treatment methods is constantly changing. For common cancers, our treatment methods are still based on conventional treatment methods, such as chemotherapy, radiotherapy, and targeted drug therapy. Nevertheless, the emergence of tumor resistance has a negative impact on treatment. Regulated cell death is a gene-regulated mode of programmed cell death. After receiving specific signal transduction, cells change their physical and chemical properties and the extracellular microenvironment, resulting in structural destruction and decomposition. As research accumulates, we now know that by precisely inducing specific cell death patterns, we can treat cancer with less collateral damage than other treatments. Many newly discovered types of RCD are thought to be useful for cancer treatment. However, some experimental results suggest that some RCDs are not sensitive to cancer cell death, and some may even promote cancer progression. This review summarizes the discovered types of RCDs, reviews their clinical efficacy in cancer treatment, explores their anticancer mechanisms, and discusses the feasibility of some newly discovered RCDs for cancer treatment in combination with the immune and tumor microenvironment.

## Introduction

With the increase in the incidence of various types of cancer such as breast, kidney, and lung cancers, cancer therapy has always been the focus of clinical development (1). With the improvement of the tumor gene spectrum, cancer treatment has been developed from early radiotherapy and chemotherapy to targeted therapy, immunotherapy, and other personalized therapeutic approaches (2). The earlier methods mainly focused on preventing the biosynthesis of cancer cells and reducing their ability to reproduce and metastasize. As research has progressed, many researchers have realized that promoting the death of cancer cells is also a feasible way to treat cancer (3). Cell death can be classified into accidental cell death (ACD) and regulated cell death (RCD) (4). ACD is generally unregulated and usually results from detrimental stimuli that exceed the cell’s ability to control. RCD is defined as programmed cell death (PCD) and is generally regulated by signaling pathways (5). Since apoptosis was discovered in 1972, more than 15 types of RCDs have been unraveled by researchers (5, 6). The Nomenclature Committee on Cell Death (NCCD), updated in 2018, has formulated the current classification, interpretations in addition to the morphological, biochemical, and functional definitions of cell death (7). RCD has been widely studied in the field of cancer treatment, including apoptosis, necroptosis, and other forms of RCD. Additionally, it has been proven to be feasible for guiding the new direction of cancer treatment (8). Because of their different molecular mechanisms, different types of RCDs can often be used as therapeutic alternatives to each other (9). Although the positive role of RCD in cancer treatment is well established, it is still a double-edged sword as some studies have shown that the RCD mechanism can also be utilized to promote tumor growth (10). Therefore, the selective manipulation of RCD to treat cancer is the focus of current research. In some cancers related to lipid accumulation, such as renal and breast cancers, lipid metabolism often regulates cancer development (11–13). Many studies have shown that lipid metabolism is closely associated with some RCDs, such as apoptosis and ferroptosis (14–16). Therefore, targeting lipid metabolism to induce cancer cell death in cancers that are sensitive to lipid metabolism can be an adequate therapeutic approach. In addition, some RCDs such as immunogenic cell death (ICD) have been considered to be associated with immunology (17). This review introduces different kinds of RCDs, examines their relationship with each other on one hand and their relationship with regards to cancer occurrence and development on the other hand. Additionally, this review discusses the possibility of application of various RCDs including lipid metabolism and cellular immunity in cancer treatment.

## Classical RCD

Based on the functional differences, there are two main types of cell death: ACD and RCD (6). ACD is an uncontrolled mode of cell death triggered by external detrimental stimuli due to the inability of the affected cells to respond beyond their regulatory capacity (18). RCD is a cell death pathway regulated by genes or signaling molecules and involves a signaling cascade by effector molecules. RCD generally has unique biochemical and morphological characteristics as well as immunological consequences (19).

Since the discovery of apoptosis in 1972 (20, 21), research on RCD has shown continuous progress. By 2018, more than 10 different types of RCD were identified. These include necroptosis, pyroptosis, ferroptosis, parthanatos, immunogenic cell death (ICD), lysosome-dependent cell death (LCD), necrotic cell death (NCD), and autophagy-dependent cell death (5).

### Apoptosis and Caspase Family

**Figure 1:**
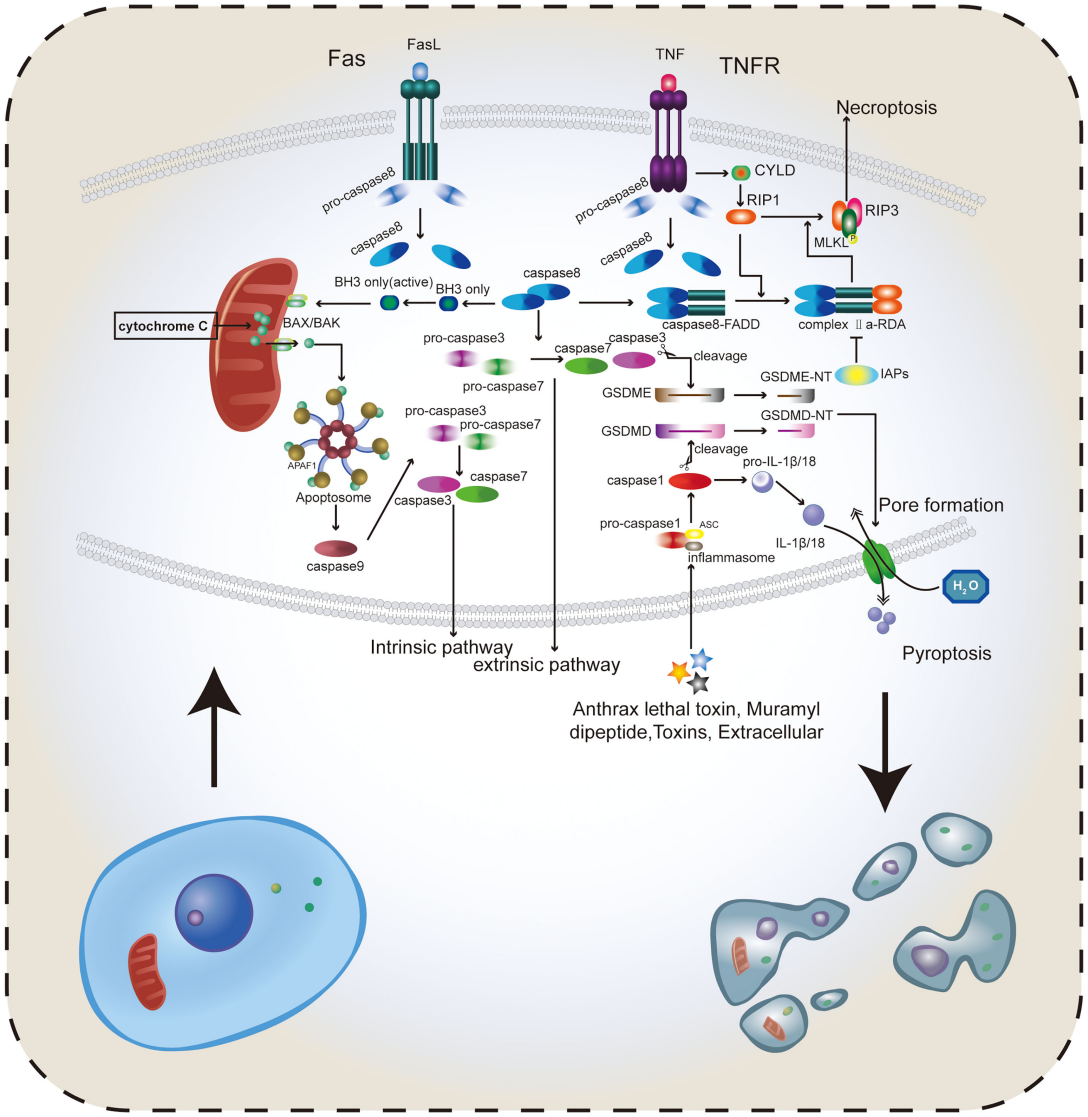
Several important types of RCD. Apoptosis includes both intrinsic and extrinsic pathways. The intrinsic pathway mainly depends on caspase-8 to activate BAX/BAK in the mitochondria, release cytochrome C, and promote the activation of pro-caspase-3 and pro-caspase-7 forming caspase-3 and caspase-7. In the extrinsic pathway, caspase-8 directly promotes the formation of caspase-3 and caspase-7, thus inducing apoptosis. Activated caspase-3 and caspase-7 can cleave GSDMD and GSDME to form GSDMD-NT and GSDME-NT, which can be adsorbed on the cell membrane and create holes, thus destroying the intracellular environmental homeostasis and inducing pyroptosis. TNF binding to receptors can activate CYLD and promote RIP1, RIP3, and MLKL to form trimers. Caspase-8 and the dimer formed by FADD in FAS bind to RIP1 to form complex IIa-RDA, which promotes this binding reaction and ultimately RIP1-RIP3-MLKL trimer binds to the cell membrane to induce necroptosis. The caspase family plays a major role in the pathogenesis of apoptosis, necroptosis, and pyroptosis. Apoptosis and necroptosis tend to change the cell membrane structure and intracellular physical and chemical properties, and pyroptosis can directly drill into the cell membrane through the GSDM protein, destroying the integrity of the cell membrane. Therefore, reasonably inducing the occurrence of these kinds of RCDs can effectively eradicate cancer cells. Caspase-8 plays an important role in caspase-dependent RCD.

Apoptosis is an active programmed cell death caused by gene regulation that maintains homeostasis in the internal environment. The apoptosis activation pathway is diverse, including intrinsic and extrinsic pathways among others. These pathways will eventually promote the cysteinyl aspartate specific proteinase (caspase) cascade reaction; thus, apoptosis depends on the caspase family (22). The extrinsic pathway can be activated by binding of receptors, including type 1 TNF receptor (TNFR1) and related protein Fas (CD95) to their ligands, TNF and Fas ligand (FasL), respectively (23). When the intrinsic pathway is activated, the Bcl-2 pro-apoptotic protein family (Bax, Bak, BID, PUMA, etc.) is activated, releasing cytochrome C from the mitochondria. Cytochrome C then binds to apoptotic protease activating factor-1 (APAF-1), forming a polymer that activates and binds to caspase-9 forming apoptotic bodies (24). Activated caspase-9, in turn, activates caspase-3, which can shear poly (ADP-ribose) polymerase (PARP) that normally exhibits a negative regulation on the endonuclease activity. This results in increasing the endonuclease activity and DNA cleavage (25). The initiator caspase-3 and the effector caspase-9 play roles upstream and downstream of the apoptosis signaling pathway, respectively (22, 26). Fourteen different members of the caspase family were identified. Most caspases directly participate in the apoptosis process, while only a few caspases participate indirectly. The caspases involved in signal transduction not only affect apoptosis, but also affect a variety of RCDS, including pyroptosis and anoikis (27), in addition to affecting inflammation (27) (**Figure 1**). This review summarizes the members of the caspase family and their roles based on their relative importance (**Table 1**).

**Table 1:** The role and mechanism of Caspase family in Apoptosis and Pyroptosis.

Caspase	Main functional classification	Mechanism in apoptosis	GSDMs processing in pyroptosis	Refs
Caspase1	Inflammatory	–	GSDMD	(28)
Caspase2	Apoptosis	initiator	-	(18)
Caspase3	Apoptosis	effector	GSDME, GSDMD, GSDMB	(27, 29)
Caspase4	Inflammatory	-	GSDMD	(30)
Caspase5	Inflammatory	–	GSDMD	(30)
Caspase6	Apoptosis	effector	GSDME, GSDMD, GSDMB	(27, 31)
Caspase7	Apoptosis	effector	GSDMB	(26, 27)
Caspase8	Apoptosis	initiator	GSDME	(32)
Caspase9	Apoptosis	initiator	–	(23, 24, 27)
Caspase10	Apoptosis	initiator	-	(27)
Caspase11	Inflammatory	–	GSDMD	(30)
Caspase12	Inflammatory	-	-	(27, 30)
Caspase13	Not found in human	–	–	–
Caspase14	Not found in human	**-**	**-**	**-**

### Apoptosis in Cancer

In normal cells, detection of irreversible DNA damage can induce apoptosis. However, cells having an overexpression of the apoptosis inhibitor Bcl-2 (33) or p53 defects (34) do not undergo apoptosis and pass on these DNA mutations during cell division, leading to the accumulation of mutations and thus contributing to the occurrence of cancer. The current mainstream cancer therapy targeting apoptosis are the drugs targeting Bcl-2 family proteins, including Oblimersen sodium (Genasense Bcl-2 antisense oligonucleotide (35)) (36), inhibitors of Bcl-2 family (37), BH3 mimetics (38), and others. Silencing the anti-apoptotic Bcl family proteins/genes has also shown a promising therapeutic effect. Studies have shown that Bcl-2-specific siRNA can effectively inhibit the proliferation and promote apoptosis of pancreatic cancer cells (39). Moreover, silencing Bmi-1 expression in breast cancer cells can also promote apoptosis by down-regulating Bcl-2 expression (40). Studies have confirmed the feasibility of using the regulatory miR-15/16 of Bcl-2-associated X protein (BAX)/Bcl-2 homologous antagonist killer (BAK)-dependent pathway, the classical pathway of Bcl-2, to fight cancer (41). On the other hand, p53 plays a main role in ferroptosis (42) as discussed in this review. In general, the mechanisms by which tumor cells evade apoptosis can be roughly divided into 1. the balance of pro-apoptotic protein and anti-apoptotic protein is disrupted, 2. the function of caspase is reduced, 3. the death receptor signal is impaired (22).

#### Necroptosis

Apoptosis has conventionally been considered the only form of RCD, while necrosis has been considered a type of accidental death that is not regulated by molecular events. This misconception was later amended when necroptosis was discovered. It is a type of RCD resembling apoptosis in mechanism and necrosis in appearance (9). Necroptosis is a caspase-independent form of RCD that is driven by the activation of receptor-interacting protein kinases (RIPs) and mixed lineage kinase domain-like (MLKL) (43–45). The TNFα receptor superfamily, T cell receptors (TCRs), interferon receptors (IFNRs), and toll-like receptors (TLRs) bind to their ligands to initiate the necroptosis signaling process (8). For example, cylindromatosis (CYLD) is stimulated when the TNF-α binds to its receptor, the TNF-α receptor (46). RIP1 is then activated by CYLD to trigger the phosphorylation of RIP3 and MLKL. The complex formed by RIP1, RIP3, and MLKL, known as the necrosome or RIP1-RIP3-MLKL complex (47), regulates the transfer of the phosphorylated MLKL trimer to the plasma membrane, resulting in increased permeability of necrotic membranes (48). In addition to changing the osmotic pressure of the cell, necrotic bodies can also cause cell death by regulating ROS balance and intracellular ATP content (45). Moreover, a previous study reported that dimerization of RIP1 can lead to self-activation, followed by binding to the caspase-8–FADD complex to form complex IIa-RDA (RIP1-dependent apoptosis) (49). When caspase-8 is inactivated or inhibitors of apoptosis proteins (IAPs) are inhibited, RIP1 binds RIP3 and MLKL to form a necrosome, leading to necroptosis (3, 44, 50) (**Figure 1**). This shows that caspase-8 plays an important role in both apoptosis and necroptosis (51). A methylation study has shown that RIP3 expression is absent in cancer cells (52). Many key necroptosis factors are downregulated in cancer, including RIPs, MLKL, CYLD, and FADD. Stoll et al. (53) reported the downregulation of RIP3 in breast cancer leading to a poor prognosis. Other studies have showed that the upregulation of RIP1 in lung cancer can reduce the ROS, thus promoting oncogenesis (54). However, in head and neck squamous cell carcinoma, RIP1 was found to be downregulated and was correlated with enhanced tumorigenesis (55). Decreased CYLD expression found in melanoma and chronic lymphocytic cells can enhance tumor progression and reduce OS in patients (56, 57). Research has also shown that decreased MLKL expression decreases the overall survival and leads to poor prognosis in different types of cancer (58–60).

Apoptosis and necroptosis, the first two forms of RCD discovered, have been playing an active role in cancer. The development of various new small-molecule drugs is aimed at activating the apoptotic pathway of cancer cells, thereby promoting cell death. The current mainstream direction points to the gene-level regulation of cellular apoptosis and necroptosis, and inhibiting or overexpressing the expression levels of related genes promotes the occurrence of cellular apoptosis and necroptosis. Not only these two RCDs, but more newly discovered RCD types have also been confirmed to be applicable in cancer treatment.

## Caspase-Dependent RCD in Target Therapy

### Anoikis and Autophagy

Anoikis was first discovered in 1993 and explains the susceptibility of outlier cells to death (61, 62). Apoptosis occurs when tumor cells detach from the primary site and spread through the circulatory system owing to the biological relevance and function of anoikis (63). Anoikis includes endogenous and exogenous pathways. In the endogenous pathway, normal aggregation of cells can come in contact with the extracellular matrix (ECM), which in turn can activate the pro-apoptotic factor Bim/Bid, synthesize Bax/Bak oligomers, inhibit the anti-apoptotic protein Bcl-XL, and eventually induce apoptosis (64, 65). In the exogenous pathway, the Fas ligand and TNF receptor apoptosis-inducing ligand (TRAIL) and their corresponding receptors bind to polymerized receptors to recruit and activate the adaptor protein Fas-related death domain (FADD). Afterward, the death effect domain of FADD binds to the death receptor to recruit caspase-8, resulting in the formation of the death-inducing signal complex object (DISC). This in turn promotes the dimerization, activation, and cleavage of caspase-8, which is then released to the cytoplasm, activating the effector molecules caspase-3 and caspase-7. Effector caspases cleave and degrade different cellular proteins eventually leading to apoptosis. FADD-induced activation of caspase-8 can also activate and dissociate Bid, destroy the mitochondrial membrane, and lead to apoptosis (8, 66, 67). There is a less common pathway in which AES/TLE1 heterooligomers are translocated from the nucleus to the cytoplasm after cells lose contact with the ECM. Subsequent proteasomal downregulation of TLE1 leads to the activation of the death signaling pathway (63). Anoikis is thought to occur when cancer cells are detached and lose contact with the ECM. However, tumor cells may resist anoikis through mutation-induced secretion abnormalities after they lose the intercellular and ECM contact. Constructional activation of pro-survival signals such as PI3K, RAS-ERK, NF-κB, and Rho GTPase occurs frequently in cancer cells and antagonizes anoikis (68, 69). Autophagy has also been found to protect cancer cells from anoikis (70, 71). Therefore, treatment approaches targeting the inhibition of cancer cells’ resistance to anoikis is currently the mainstream research strategy.

Cancer cells acquire resistance against anoikis by regulating integrins and activating the EMT. Integrins play a key role in regulating cell contact with the ECM (72). Integrins are bidirectional signaling molecules with two different conformational states that determine their different affinities for the ECM. Closed integrins have a low affinity for ECM ligands, while open integrins have a high affinity for ECM and bind to ECM to induce downstream signal transduction (73). Previously, integrins and their cancer-regulating functions were thought to be limited to signal transduction in plasma membranes and focal adhesions. The loss of ECM tension during matrix degradation was suggested to allow the uptake of active integrin-binding ligand fragments. This explains why ECM ligands, such as active integrins, are easily detected in the endosomes of cancer cells (73, 74). Epithelial-mesenchymal transition (EMT) promotes epithelial cancer cells to acquire mesenchymal characteristics through inhibiting epithelial cell markers and upregulating mesenchymal markers. This facilitates the metastasis of cancer cells and is an important resistance mechanism of cancer cells to anoikis (75).

Autophagy, a type of cell death that is completely varies from apoptosis, has been recognized to have a dual role where it can either inhibit or promote cancer metastasis (8). The anti-metastatic effect of autophagy is mainly through four mechanisms. The first mechanism is through decreasing tumor necrosis induced by hypoxia and preventing the infiltration of inflammatory cells. The second mechanism is through regulating the release of HMGB1 from cancer cells, thus mediating the anticancer immune response. The third mechanism of autophagy involves the direct death of cancer cells. The last mechanism is through triggering apoptosis, thus resulting in cancer cell death (76). On the other hand, autophagy promotes cancer metastasis and has been linked to anoikis. Research has shown that autophagy provides a mechanism for stromal isolated premetastatic tumor cells to resist anoikis (71). In a hepatocellular carcinoma metastasis model, inhibition of autophagy did not affect cell invasion, migration, or EMT, but attenuated the anoikis resistance of hepatocellular carcinoma cells. This greatly enhanced the ability of hepatocellular carcinoma to metastasize (77). Similarly, other studies have shown that ECM detachment and β1 integrin inhibition can induce autophagy (78). Experiments have shown that there is a correlation between autophagy and anoikis.

Cancer treatment for anoikis are becoming available, and more *in vitro* and *in vivo* trials are supporting this treatment strategy. Studies have shown that carnitine palmityl transferase 1A (CPT1A)-mediated fatty acid oxidation (FAO) can help colon cancer cells resist anoikis and thus promote colon cancer metastasis. Researchers have identified CPT1A as a potential target for colon cancer treatment (79). Myosin heavy chain 9 (MYH9) has also been found to promote the transcription of catenin beta 1 (CTNNB1), thus rendering resistance to gastric cancer cells against anoikis both *in vivo* and *in vitro* (69). Anoikis has also been employed for the treatment of lung cancer. Researchers have found that the PLAG1-GDH1 axis improves the resistance of lung cancer cells against anoikis. Glutamate dehydrogenase 1 (GDH-1) can be upregulated by pleomorphic adenoma gene 1 (PLAG1), and its product, α-KG, activates calcium/calmodulin-dependent protein kinase kinase 2 (CamKK2) by enhancing the binding of CamKK2 to the substrate adenosine 5’-monophosphate (AMP)-activated protein kinase (AMPK). This binding contributes to energy production and thus to resistance against anoikis (80). Jin et al. showed that lactate dehydrogenase A (LDHA), an enzyme that catalyzes the conversion of pyruvate to lactate, is phosphorylated at tyrosine 10 by upstream kinases HER2 and Src, thus promoting anoikis resistance in breast cancer (81).

### Pyroptosis

Pyroptosis, a type of RCD generally caused by the inflammasome, is mainly characterized by the expansion of the cells until the membrane is ruptured and the cellular contents overflow, thus inducing a potent inflammatory response (82). The occurrence of pyroptosis depends on the caspase family and the GSDM protein family. After the activated caspase cleaves the GSDM protein, the released GSDM n-terminal (GSDM-NT) (83) binds to and drills in the cell membrane, resulting in changes in the cellular osmotic pressure, cellular swelling, and eventually cell rupture (82, 84).

Currently, there are three main pathways of pyroptosis, namely the canonical inflammasome pathway, the non-canonical inflammasome pathway, and the extracellular fluid pathway (85). Caspases involved in these pathways include caspases-1, 4, 5, and 11 (85, 86). The canonical inflammasome pathway has a relatively clear mechanism. Inflammasomes in pyroptosis, including NLRP1, NLRP3, NLRC4, and AIM2, are usually activated by pathogens and their secretions such as thymodyl dipeptide, flagellin, and double-stranded DNA (dsDNA) (87–89) (**Table 2**). NLRP3, in particular, is activated by a wide range of stimuli, including ROS, extracellular RNA, uric acid, and cholesterol (**Table 2**) (90–92). The activated inflammasome combines with apoptosis-associated speck-like protein containing a CARD (ASC) and recruits procaspase-1, which activates caspase-1 (93, 94). Caspase-1 cleaves GSDMD and releases GSDMD-NT into the membrane, which leads to membrane drilling (95). Caspase 1 also cleaves proL-18 and proIL-1, which participate in the maturation of proIL-18 and proIL-1 β, which are in turn released into the extracellular space and trigger inflammatory responses (96, 97) (**Figure 1**). Pyroptosis is an extensive frontier of cancer. Current studies report that the role of pyroptosis in cancer cells is complicated; thus, the deployment of pyroptosis to combat cancer has always been a research focus area. In esophageal squamous cell carcinoma (ESCC), BI2536, an inhibitor of polo-like kinase 1(PLK1), was found to activate caspase-3 and BAX in combination with cisplatin, resulting in GSDME disruption and increased DNA damage. Meanwhile, GSDME was also found to be highly expressed in ESCC, suggesting that BI2536 can markedly increase the sensitivity of ESCC to chemotherapy (98). In gastric cancer (GC), the low expression of pyroptosis-affecting protein, GSDMD, is one of the reasons for promoting the proliferation of cancer cells. GSDMD was found to reduce the expression of cyclin A2 and cyclin-dependent kinase 2 (CDK2), which slowed down DNA synthesis and S-G2 phase progression in cyclin through ERK1/2, STAT3, and PI3K/AKT (99, 100), and ultimately slowed down GC cell proliferation (101, 102). In triple-negative breast cancer (TNBC), docosahexaenoic acid (DHA, an omega-3 fatty acid) induces caspase-1 activation, which leads to GSDMD division, secretion of IL-1β, and membrane pore formation, thus promoting cancer cell death (103). HMGB1, an important damage-associated molecular pattern (DAMP), is transferred from the nucleus to the cytoplasm after DHA-induced activation of caspase-1, and facilitates the progression of pyroptosis (104). A recent study on the role of NPD-L1 and TNFα in breast cancer showed that under hypoxia, PD-L1 can migrate to the nucleus and activate GSDMC, which is then cleaved by the TNFα activated-caspase-8, triggering pyroptosis and promoting the death of breast cancer cells (105). The current mainstream view of the non-canonical inflammasome pathway is that lipopolysaccharide (LPS) drives caspase-4, 5, and 11 to cleave GSDMD, thereby triggering pyroptosis (5). The main differences between the canonical and non-canonical inflammasome pathways are the activation of caspases and the types of caspases that cleave GSDMD. In addition to GSDMC, GSDMD, a new pyroptosis pathway was reported in recent studies. GSDMD cleaves GSDME by caspase-3 to form GSDME-NT, thus enabling it to perform its function (29). Unfortunately, the unique application of this mechanism in cancer has not been found, and it cannot be differentiated from other GSDMs.

**Table 2:** Different types of Inflammasome activate Necroptosis.

Inflammasome	Primary activator	Refs
NLRP1	Anthrax lethal toxin, Muramyl dipeptide	(87)
NLRP3	Toxins, Extracellular RNA	(90–92)
NLRC4	Flagellin, Muramyl dipeptide	(88)
AIM2	dsDNA	(93)

**Table 3:** Differences between different types of RCDS.

RCD	Major biomarkers	Characteristics of cell death	Role in cancer
Apoptosis	Caspase3,7,8,9 BAX/BAK	Apoptotic bodies form	Apoptosis promotes cancer cell death
Necroptosis	RIPK1,3 MLKL	The insertion of phosphatidylinositol into the plasma membrane results in increased plasma membrane permeability	Necroptosis promotes cancer cell death
Pyroptosis	Caspase1,4,5,11 GSDMD, GSDME	Drill holes on cell membranes	Commonly associated with cancer cell death, but may promote cancer progression in some types of cancer
Anoikis	ECM	Refer to the apoptosis	Inhibit cancer cell metastasis
Immunogenic cell death	ATP, Calreticulin, HMGB1, HSP	The cell lyses to present antigens	Chemotherapy induces cell death in time and has inhibitory effect on cancer
Ferroptosis	ACSL4, LPCAT3, ALOX15, SLC7A11, GPX4, NFE2L2	Mitochondrial damage, ruptures	It has different effects on different cancers, mainly depending on the related factors that promote the occurrence of ferroptosis, such as the level of ROS in cancer cells
Parthanatos	PARP1, AIFM1	Chromosomal dissolution	PARP1 inhibitors are well-established anti-cancer drugs and have excellent inhibitory effects on cancer
NETosis	NADPH, PAD4	Chromatin deconcentrates, the nuclear membrane destroys and chromatin fibers releases	There are multiple effects, both inhibiting cancer and causing great damage to the body as it progresses

**Figure 2:**
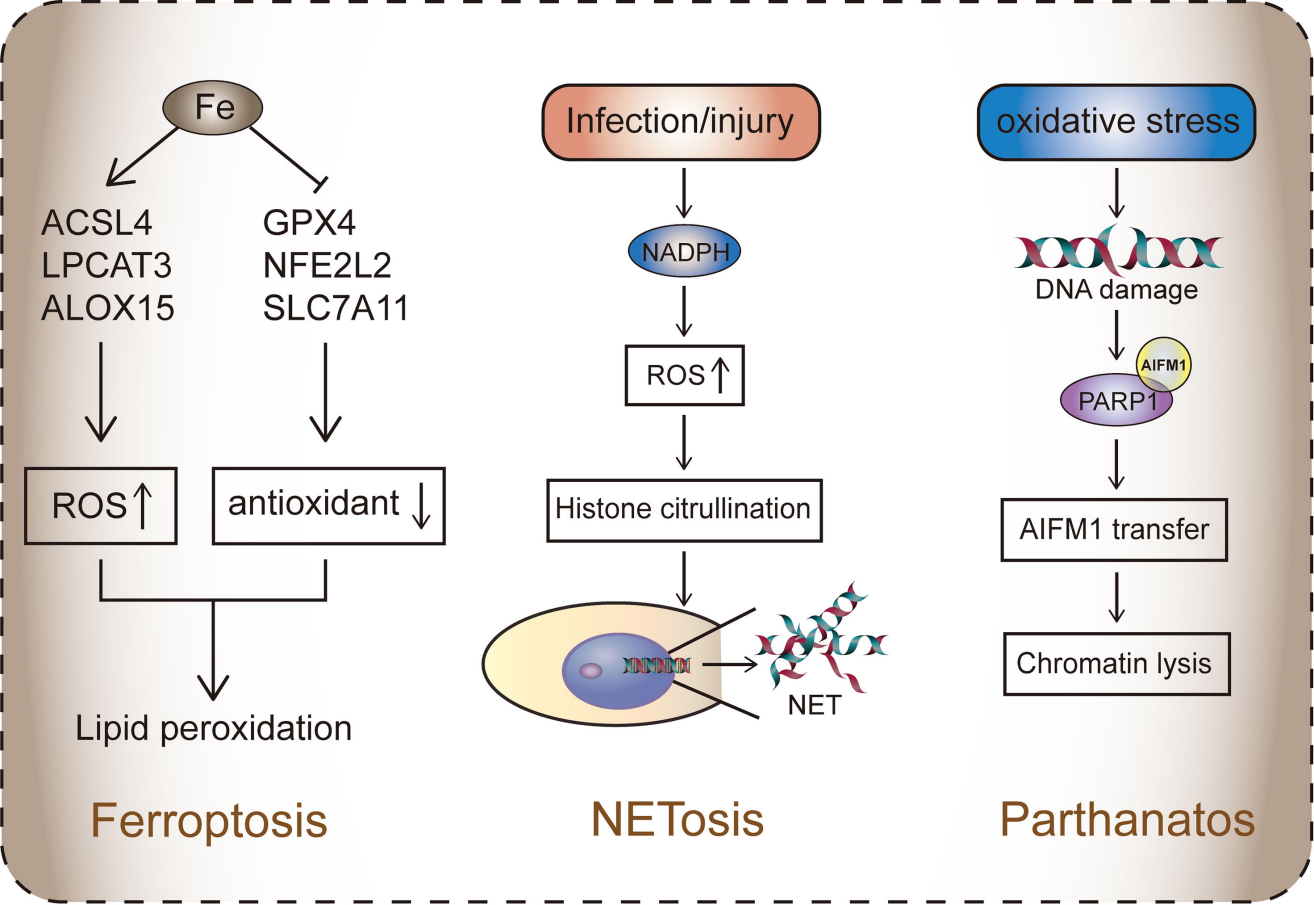
The three RCDs that do not rely on caspase. Ferroptosis is caused by the accumulation of iron, which is activated by the disruption of the balance between ROS and the antioxidant system. ACSL4, LPCAT3, ALOX15, and other genes mediate fatty acid oxidation, resulting in lipid toxicity in cells. Antioxidant systems such as Xc-, GPX4, and NFE2L2 protect cells from ROS. Ferroptosis is mainly dependent on the formation of PUFA-OOH. In system Xc-, GPX4 participates in the reduction of lipid peroxides (such as PUFA-OOH) and inhibits ferroptosis. In the lipid metabolism pathway, lipid droplets decompose to PUFA and AA/AdA. The latter is processed by ACSL4-LPCAT3-ALOX15 to form PUFA-OOH.NETosis: ROS mediated by NADPH activates histone citrullination, leading to the release of NET (chromatin in the nucleus), which blocks invading substances such as pathogens.Parthanatos: Oxidative stress leads to DNA damage. Activated PARP1 binds to AIFM1, causing the latter to migrate to the nucleus, leading to the dissolution of part of the chromosome.

To date, the role of pyroptosis in cancer is still not fully understood. Research studies on the signaling pathways related to the pyroptosis pathway are relatively limited; therefore, more studies and analyses are needed to confirm its effect.

From a molecular mechanism perspective, the main difference between apoptosis, necroptosis, and pyroptosis depends on the caspase family members they activate. This also implies an inter-relationship between the three caspase-dependent RCDs. Therefore, the combined effects of those pathways must be considered in studies involving cancer treatment approaches.

## Caspase-Independent RCD in Targeted Therapy

The discovery of caspase and GSDM proteins promoted RCD-related research. However, many other RCD types can also exercise their functions without these two proteins. The imbalance of various cytokines, proteins, and cholesterol contained in the cancer cell microenvironment may cause cell death. Therefore, we reviewed other non-caspase/GSDM-dependent RCDs.

### Ferroptosis

Ferroptosis depends on the balance between ROS, resulting from lipid peroxidation due to iron accumulation, and the antioxidant system. Lipid peroxidation results in mitochondrial diminution, mitochondrial crest reduction, increased membrane density, and membrane rupture (106). The pathways affecting iron-mediated death include GSH/GPX4 pathway, iron metabolism pathway, and lipid metabolism pathway (107).

The Xc- system is an amino acid anti-transporter located in cell membranes and is part of an important antioxidant system in cells. It is a heterodimer composed of two subunits, SLC7A11 and SLC3A2 (108). Xc- regulates the exchange of cysteine and glutamate. The ingested cystine is reduced in the cells to cysteine, which is involved in glutathione (GSH) synthesis. GSH reduces ROS and reactive nitrogen in the presence of glutathione peroxidase (GPX) (109). Therefore, when systemic Xc- inhibition occurs, the antioxidant capacity of cells decreases, and ROS accumulation eventually leads to ferroptosis (110). P53 can also inhibit the absorption of cystine by downregulating the expression of SLC7A11 through system Xc-, thus affecting GPX4 activity (42) (**Figure 2**).

VDACs are transmembrane transport channels of ions and metabolites that play an important regulatory role in ferroptosis (111). Erastin was found to act on VDACs, causing mitochondrial dysfunction and the release of large amounts of ROS, ultimately leading to iron-mediated ferroptosis (112). TF receptor 1 (TFR1) promotes iron absorption and increases the intracellular concentration of iron, which promotes iron-mediated ferroptosis (113). A study reported that overexpression of heat shock protein beta-1 (HSPB1) can significantly inhibit ferroptosis, mainly due to the inhibition of TFR1 membrane protein by HSPB1 (114).

Almost all the regulatory pathways of ferroptosis is dependent on ROS produced by lipid peroxidation (106). Polyunsaturated fatty acids (PUFAs) are sensitive to lipid peroxidation and are key substances in the mechanism of ferroptosis. The esterification and oxidation products of PUFAs, arachidonic acid (AA) and phosphatidylethanolamine (PE), can transmit ferroptosis signals and promote ferroptosis (115). Acyl-CoA synthase long-chain family member 4 (ACSL4) and lysophosphatidyl cholinyl transferase 3 (LPCAT3) participate in PE biosynthesis and remodeling, activate PUFAs, and affect the transmembrane properties of PUFAs. PUFA-PE is further oxidized by lipoxygenase (LOX) and eventually induces ferroptosis (116). Therefore, lowering the expression of ACSL4 and LPCAT3 can reduce the accumulation of lipid peroxide substrates in cells, thereby inhibiting ferroptosis.

Since ferroptosis has a special focus in cancer therapy, our team identified a large number of ferroptosis pathway genes that are highly expressed in various cancer patients through bioinformatics analysis (117). The gene expression of some of the proteins (such as ACSL4, SLC7A11, and ALOX15) is altered in different cancers. It is worth noting that our study found that ferroptosis often plays a dual role in tumor progression. This phenomenon is thought to be influenced by the balance between the release of damage-associated molecular patterns and the immune response induced by ferroptosis (118).

The P62-KEAP1-NrF2 pathway plays a key role in ferroptosis in hepatocellular carcinoma cells. P62 can destroy the structure of Keap1 and attenuate its degradation of NRF2, resulting in NRF2 accumulation in cells (119). NRF2 inhibits ferroptosis by upregulating quinone oxidoreductase 1(NQO1), heme oxygenase-1 (HO-1), and ferritin heavy chain 1(FTH1), thus promoting iron and ROS metabolism (120). Inhibition of NRF2 expression either by genetic tools or drugs significantly enhanced the antitumor effects of erastin and sorafenib in HCC, while activation of NRF2 expression resulted in hepatocellular carcinoma resistance to ferroptosis (121). Serramazine and lapatinib significantly increased iron-dependent ROS levels in breast cancer cells (122). However, cysteine dioxygenase type 1 (CDO1) overexpression can reduce GSH expression and ROS accumulation in breast cancer cells (123). Therefore, studies have shown that these two drugs have a better therapeutic effect in CDO1 overexpressing breast cancer cells (124).

This was also observed in clear cell renal carcinoma (ccRCC). Bioinformatics analysis showed that CDO1 promoter methylation was significantly correlated with poor prognosis of ccRCC patients, suggesting that CDO1 promoter methylation may be a new prognostic molecular marker of ccRCC (125). Studies have shown that p53 can prevent the aggregation of dipeptidyl peptidase-4 (DDP4) on the plasma membrane, weaken lipid peroxidation, and ultimately lead to ferroptosis (126). In colorectal cancer, p53 inhibits DDP4 activity, resulting in cancer cell resistance to ferroptosis. Our bioinformatics analysis showed that, as the core of ferroptosis, mutations in ROS-induced oxidative stress pathway-related regulatory genes are widely present in more than thirty types of cancer, and their high expression in ccRCC leads to a good prognosis in patients (127).

According to the current research, the development of anticancer drugs based on ferroptosis mainly focuses on two aspects; system Xc and GPX4. The survival and growth of cancer cells strongly depend on the transport activity of system Xc-, making system Xc- a potential target for anticancer drug development.

Systemic Xc- inhibitors can inhibit cystine uptake and interfere with cellular mechanisms that control protein folding, induce cellular stress, and thus lead to ferroptosis (128). Erastins is a prototype ferroptosis inducer that can directly inhibit system Xc-. Erastin has been shown to activate ferroptosis in tumor cells by upregulating the RAF/MEK/ERK signaling pathway (112, 129). It has also been shown to enhance the effectiveness of some traditional anticancer drugs in certain cases, such as doxorubicin (130) and cisplatin (131). Other studies have shown that erastin resistance can be induced by the knockout of its target gene, the voltage-dependent anion channel 2/3(VDAC) (132). Imidazole ketone Erastin, an Erastin derivative, has been successfully applied in heterogeneous animal models for the treatment of diffuse large B-cell lymphoma (DLBCL) owing to its excellent administration efficiency and anticancer performance (133).

Sorafenib is a clinically approved multikinase inhibitor for the treatment of advanced cancers, such as renal and liver cancers (134, 135). Research shows that sorafenib may inhibit systemic Xc by two potential mechanisms, which are either through inactivating kinases necessary for systemic Xc activity or interactions with non-kinase targets and their binding sites, which are similar to those of sorafenib-sensitive kinases (136). Several tumors are currently resistant to Sorafenib. Metallothionein-1g (MT-1G), a transcription target of the redox regulator NRF2, has been observed in drug-resistant cancer cells. Metallothioneins protect cells from the oxidative damage caused by heavy metals through binding to them. Therefore, inhibition of the MT-1G pathway during sorafenib treatment can reduce the risk of chemotherapy resistance and improve its therapeutic effect (137).

It is worth noting that some cancer cells can bypass system Xc- and synthesize cysteine through the transsulfation pathway. This suggests that inhibition of system Xc- therapy is not suitable for all cancers. GPX4 is a central regulator of iron function, and its inactivation leads to iron function loss, even at normal cysteine and GSH levels (138).

RSL3 targets enzymes with nucleophilic sites (e.g., cysteine serine selenocysteine) and inactivates GPX4 directly by alkylation of selenocysteine (139). One of the four diastereomers of RSL3, namely (1S, 3R)-RSL3, has been found to be more selective and lethal to cancer cells; thus, it is considered to be the optimal scheme for the application of RSL3 in cancer therapy (140). Ghoochani’s studies confirmed that prostate cancer cells were sensitive to (1S, 3R)-RSL3 and suggested that RSL3 could induce ferroptosis in tumor cells (141).

FIN56 is a ferroptosis inducer derived from CIL56. Compared with CIL56, FIN56 has a higher efficiency and specificity in inducing ferroptosis. FIN56 promotes GPX4 degradation, which requires the enzymatic activity of acetyl-CoA carboxylase (ACC) and simultaneously binds and activates squalene synthetase (SQS), leading to the depletion of the endogenous antioxidant CoQ10 and enhanced cell sensitivity to fin56-induced ferroptosis (142). Sun et al. showed that FIN56 can induce ferroptosis in bladder cancer cells and can be enhanced by combination with the mTOR inhibitor, Torin 2 (143). Zhang et al. reported that FIN56 can increase lysosomal membrane permeability through a tFeb-dependent pathway and thus promote glioblastoma cell death (144).

Since its discovery in 2012, ferroptosis has been widely studied as a therapeutic approach for the treatment of cancer; thus, its anticancer mechanism is worth further exploration.

### Parthanatos and PARP Inhibitors

Parthanatos is a PARP1-dependent mode of cell death that is typically caused by poly ADP-ribose Polymerase-1 (PARP1) overactivation. PARP is a multifunctional post-translational modification enzyme that is present in most eukaryotic cells. It is activated by the recognition of structurally damaged DNA fragments and then performs DNA repair (145). PARP primarily transfers ADP-ribose units from nicotinamide adenine dinucleotides to receptor proteins, including histones, RNA polymerase, DNA polymerase, and DNA ligase (146). Under pathophysiological conditions, overactivation of PARP1 is usually caused by DNA damage, leading to the accumulation of poly ADP-Ribose (PAR) and the nuclear translocation of apoptosis-inducing factor (AIF). This in turn leads to the dissolution of part of the chromosome, ultimately triggering parthanatos (147). DNA damage that triggers PARP1 activation is usually induced by ultraviolet light, reactive oxygen species (ROS), or alkylation agents. In addition, activation of the calcium (Ca^2+^) signaling pathway or DNA modification (including phosphorylation, acetylation, etc.) can also induce PARP1 activation (148, 149) (**Figure 2**). Parthanatos is involved in several important pathological processes, including ischemia-reperfusion injury after cerebral ischemia or myocardial infarction (150, 151), and neurodegenerative diseases such as Parkinson’s disease and Alzheimer’s disease (152).

Parthanatos is closely related to cancer, mainly because PARP1 plays an important role in the occurrence, development, and treatment of tumors. First, PARP1 has a dual nature, acting as both a promoter of DNA repair/replication and a stimulator of DNA fragments (153). The role of PARP1 in cancer is complicated. The loss of PARP1 often leads to impairment in the DNA repair machinery, which can contribute to cancer development (154, 155). However, inhibiting the function of PARP can also help in the treatment of cancer. The primary role of PARP inhibitors was to inhibit DNA repair and enhance the efficacy of chemotherapy and radiation. PARP inhibitors bind to the PARP1 or PARP2 catalytic site, preventing the PARP protein from shedding from the DNA damage site. This in turn leads to failure of DNA replication and activation of homologous recombination repair (HRR) as a compensatory mechanism (156). This involves a newly proposed principle of synthetic death.

Synthetic death implies that a cell dies when two genes or proteins are altered at the same time, but the cell is able to survive if only one of those genes or proteins is altered (157). BRCA1 and BRCA2 are the key HRR proteins; consequently, when this protein fails to function properly, both genes are inactivated and the cell eventually dies under the influence of synthetic death (158). However, with the discovery and application of BRCA gene mutations, high-grade serous ovarian cancer (159, 160), advanced prostate cancer (161), and pancreatic cancer (162) have been found to potentially benefit from PARP inhibitor therapy. The earliest clinically used PARP inhibitor is rucaparib in combination with the chemotherapeutic agent, temozolomide (163, 164).

With the discovery of synthetic death, rucaparib has also been shown to treat metastatic prostate cancer patients with BRCA mutations (165). After treatment with rucaparib, olaparib was also widely promoted. Trials showed that 63% of breast cancer patients with BRCA1 or BRCA2 germline mutations could benefit from olaparib treatment (166). Niraparib and talazoparib have also been used to treat patients with BRCA germline or systemic mutations (167, 168).

It took more than a decade for the first PARP inhibitor to be FDA approved through the discovery of a relationship between PARP inhibitors and BRCA synthetic death. Currently, PARP inhibitors are still widely investigated, with several clinical trials underway to be approved for cancer treatment.

## RCD in Immunotherapy

### Immunogenic Cell Death (ICD) and Tumor Microenvironment

When tumor cells die under the influence of external stimulation (i.e., chemotherapy and radiotherapy), they change from non-immunogenic to immunogenic and mediate anti-tumor immune response after tumor cell death. This phenomenon is known as immunogenic cell death (ICD) (17). The classification of ICDs is unique, where necrosis occurs only if cell death caused by pathogen invasion takes place. However, if the immune cells encounter the target cells infected by pathogens and cause the latter to die, effector T cells directly lead to cell death. Thus, in the latter case, pathogens are only the inducing factors of the target cells’ death. The immunological properties of ICD are mediated by damage-associated molecular patterns (DAMPs), which are endogenous molecules released during cell death (169). Theoretically, many intracellular substances, including cytokines and intracellular matrix, fall into the DAMP category. DAMPs, which are also called ICD-associated DAMPs, produced in the body during chemotherapy mainly include surface-exposed calreticulin (CRT), secreted ATP, released high mobility group protein B1 (HMGB1), and heat shock protein (HSP70, HSP90, etc.) (17, 118, 170). Unlike the various programmed cell deaths mentioned above, ICD is a form of cell death that occurs during chemotherapy and can increase the effectiveness of cancer treatment through cellular immunogenicity. Therefore, DAMPs can be used to enhance the effect of chemotherapy through amplifying the effect of ICD.

### High Mobility Group Protein 1 (HMGB1)

When ICD occurs, HMGB1 is released from cells. The release of HMGB1 involves in it crossing the nuclear membrane and the plasma membrane to complete the transfer of HMGB1 from the nucleus to the cytoplasm and finally to the extracellular space. Extracellular HMGB1 binds to PRRs expressed on the surface of bone marrow cells, advanced glycosylation end-product-specific receptor (AGER or RAGE), and toll-like receptor 4 (TLR4), which activate corresponding signaling pathways and promote immune response (171). HMGB1 has been confirmed to be a tumor suppressor. Kang et al. showed that HMGB1 in the pancreas highly sensitized newborn mice to carcinogenic K-Ras-driven precancerous lesions and promoted tumor metastasis and invasion (172). Other experiments have confirmed that HMGB1 released by GSDME-mediated pyroptotic epithelial cells can be involved in colitis-related colorectal cancer lesions (173). However, HMGB1 still plays a role in promoting cancer development. A study has shown that HMGB1 regulates VEGF-D to mediate the formation of cancer blood vessels, thus promoting cancer (174).

### Calreticulin (CRT)

When ICD occurs in tumor cells, CRT is exposed to the cell membrane surface and acts as an “Eat-ME” signal. This promotes dendritic cells (DCs) to engulf dead or dying tumor cells and promotes the maturation and function of DCs (175). Under stress, CRT translocates from the endoplasmic reticulum and is exposed to the membrane surface, releasing the “Eat-me” signal to activate the immune response (176). Drugs known to induce the transfer of CRT from the intracellular space to the cell membrane include anthracyclines and oxaliplatin (177). The adverse effects of mutations in the regulatory genes of CRT on ICD are of interest. Liu et al. have shown that cancer cells secrete soluble mutant CRT into the tumor microenvironment, which binds to the CRT receptor on DC cells and prevents DC cells from contacting cancer cells, thus blocking the progression of ICD (178). By using the retention using selective hooks system (RUSH) to observe the transport of CRT, Liu et al. found that CRT produced by exon 9 mutation of the CALR gene can inhibit the phagocytosis of dendritic cells (DCS) to dying cancer cells, thus reducing the effectiveness of tumor immunotherapy by chemotherapeutic drugs or PD-1 blockade (179).

### ATP

ATP is released mainly in autophagy-dependent mode during ICD and is released in the form of vesicles *via* the ANNexin channel. ICD is activated by the release of a “Find-me” signal *via* the purinergic receptor P2RY2, which is picked up by DC progenitors and macrophages. ATP released from the cell is also involved in mediating the development of proinflammatory cytokines, activating the formation of the casp1-dependent NLRP3 inflammasome and secretion of mature IL-1β and IL-18 (180, 181). This process is like the typical inflammasome pathway of pyroptosis. Therefore, the ICD process is believed to involve pyroptosis.

### iDAMPs

Recent studies have shown that DAMPs not only activate the immunogenicity of the body against cancer cells, but also many immunosuppressive DAMPs have been discovered successively, including HSP60 and adenosine (182). There is an intricate balance between immune stimulation and inhibition by DAMPs. Striking this balance is important to help some drugs that do not have an intrinsic ability to induce ICD to produce greater therapeutic efficacy. A new perspective, which categorizes immunosuppressive DAMPs as iDAMP, suggests that by blocking their effects, it is possible to transform the nature of some chemotherapeutic drugs and improve their efficacy (183). Unfortunately, there has not been much experimental confirmation of this novel idea. Recent studies have shown that gemcitabine can be transformed from being a non-ICD-mediated chemotherapeutic agent to an ICD-mediated chemotherapeutic agent through the COX-2/PGE2 pathway, which mediates a large number of CD8+ T cells into tumor tissues and enhances the anticancer efficacy of gemcitabine (184). This experiment also demonstrated that the immunosuppressive function of iDAMP is a physiological response to drug-induced cell death, which helps chemotherapeutic drugs better mediate ICD.

### Application of Nano-Vesicle Carrier Combined With Chemotherapy Drugs

Activating the anti-tumor immune response of T cells by triggering ICD is a conventional approach for the application of ICD in tumor therapy. Many conventional clinical trials based on this approach have been initiated. The current problems include low drug delivery efficiency and avoidance of immunosuppression in the tumor microenvironment. To solve these problems, the integration of chemotherapeutic drugs and polyethylene glycol photosensitizers with nano-platforms seems to be the best solution. Zhou et al. used a tumor microenvironment-activatable prodrug vesicle for cancer chemoimmunotherapy to treat cancer. Their study showed that oxaliplatin and PEGylated photosensitizers could be integrated into the same nanoplatform to effectively improve the efficiency of drug delivery and inhibit tumor immune escape by blocking CD47 (185). Another study showed that integrated ph-responsive nanovesicles(pRNVs)/2-(1-hexyloxyethyl)-2-devinyl pyropheophorbide–A(HPHH)/indoximod (IND) could produce significant anti-tumor effects in melanoma (186). It was found that HPHH-mediated photodynamic therapy (PDT) that produces singlet oxygen, combined with PRNV to induce ICD, promoted dendritic cell (DC) recruitment and increased immune response stimulation. Meanwhile, IND regulates the tumor microenvironment by promoting the development of CD8+ T cells. ICD and tumor-infiltrating T lymphocytes are severely impaired by elevated ROS levels in the tumor microenvironment. Therefore, the regulation of extracellular ROS levels is essential to reverse the immunosuppressive environment. Based on the reactivity between pRNV and ROS, Deng et al. (187) targeted the ROS removal in the tumor microenvironment by anchoring pRNV on tumor ECM, alleviated immunosuppressant ICD caused by specific chemotherapy, and extended the survival time of T cells.

In addition to anthracycline-based drugs, some of the commonly used chemotherapeutic drugs have been found to promote ICD, including bleomycin, bortezomib, cyclophosphamide, actinomycin D, and teniposide. Some drugs can only promote the secretion of CRT like bortezomib, others can promote the secretion of CRT and HMGB1 like Teniposide, while most of the drugs can induce a variety of DAMPs (181). In addition, we found that many drugs, including cyclophosphamide and teniposide, can also enhance anti-PD-1 and anti-CTLA-4 therapies. PD-1 and CTLA-4 are protein receptors located on the surface of T cells for immune regulation, The combination of PD-L1 and CD80/86 on the surface of tumors can shut down the function of T cells and prevent tumor cell death (188, 189). The PD1 ligand, PD-L1, is highly expressed in several cancers; hence, the role of PD1 in cancer immune evasion has been well established. Inhibition of PD-1 and PD-L1 interactions enhances T cell responses *in vitro* and mediates preclinical antitumor activity. This is known as immune checkpoint blockade therapy (190). Our study demonstrated the efficacy of anti-PD-1 and anti-CTLA-4 therapies in renal cancer (191). Unfortunately, widespread resistance has been found against this treatment; therefore, we believe that the appropriate combination of these ICD-promoting agents with immune checkpoint blockade therapy could provide a new therapeutic potential for this approach.

Xie et al. prepared a phenol-based ICD inducer for tumor cells *in vitro* by combining doxorubicin (DOX), phenolic manganese dioxide nanoreactor, ferric iron, and polyethylene glycol polyphenol (MDP NPs) through metal phenol coordination assembly (192). They found that MDP NPs enhanced DOX-mediated ROS-dependent cell death and accelerated ICD induction. Subsequently, MDP NPs successively lead to the enhancement of tumor-associated antigens, maturation of dendritic cells, and ultimately enhancement of tumor-specific T cell infiltration. MDP and NPs can also effectively recruit macrophages, thus improving the tumor response to PD-1 checkpoint blocking immunotherapy. This eventually resulted in a significant anti-tumor immune response.

In these studies, we found that PDT and nanoparticles are an important means of using ICD to treat cancer. However, PDT has some limitations, including a reduction in the efficiency of ICD in the hypoxic tumor microenvironment. Technology based on both PDT and nano-platforms can perfectly compensate for each other’s shortcomings without compromising their own efficacy, which renders this combined therapeutic approach high efficiency, high targeting and personalization.

### NETosis

Neutrophils play a key role in immunity. Neutrophils usually perform their functions by directly phagocytosing pathogens and secreting cytotoxic enzymes to produce neutrophil extracellular traps (NETs) (193, 194). Brinkmann et al. first discovered the NETs and their function in immunity in 2004 (195). The main structure of NETs is reticular DNA formed by depolymerized chromatin and is surrounded by a variety of nuclear proteins, including histone granular proteins and cytoplasmic proteins (196). Brinkmann et al. found that the released DNA can capture and neutralize pathogens. This process is called NETosis.

NET release begins with the activation of neutrophils, where neutrophil surface receptors bind to ligands. Studies have shown that neutrophils lacking surface receptors do not develop NETosis (197). G protein-coupled receptor ligand (GPCR), interleukin-1(IL-1), tumor necrosis factor (TNF) and Fc receptor can induce NETosis (198–200). In addition, activation of Nod-like receptor protein 3, some bacterial toxins, and ROS can also induce NETosis instead of binding to neutrophil surface receptors (201–203). There is also a theory that injury or infection induces the body’s stress state, which activates oxidative stress and causes histone citrullination by NAPDH, producing a high amount of ROS (204). The formation of NETs depends on chromatin desorption, nuclear membrane degradation, and cell lysis by peptidyl-arginine deiminase 4-mediated citrullination (conversion of arginine to citrulline). It is a type of epigenetic histone modification (205). Many studies have confirmed that PAD4 is a marker for NETosis, and the loss of PAD4 causes NETosis to fail to initiate (206, 207). Degradation of the nuclear membrane is driven by neutrophil elastase (NE), which receives a superior signal transfer to the nucleus, resulting in the rupture of the nuclear membrane (208). It is exciting that the latest research results show that NE plays a role in the destruction of nuclear membrane mechanisms, which are closely related to pyroptosis. ROS promotes NE release into the cytoplasm. NE released from granules can shear GSDMD and release GSDMD-NT, destroying the nuclear membrane and cell membrane and causing neutrophil lysis. The production of GSDMD-NT is the main reason for NET secretion into the extracellular domain (209). This study not only revealed the mechanism of NET secretion, but also provided evidence that the GSDM family plays a role in a variety of different cell death pathways as cell death executors (**Figure 2**).

The role of neutrophils in tumor progression is controversial because neutrophils have both pro-tumor and anti-tumor properties (210). The neutrophil chromatin release affects tumor growth, angiogenesis, metastasis, and immunosuppression (211). Many studies have found large concentrations of neutrophils and high expression of PAD4 in cancer tissues, including Lewis lung cancer and Ewing’s sarcoma (212, 213). Simultaneously, NETs allow detached tumor cells to attach to other tissues, promoting cancer metastasis, and the by-products of NET decomposition can also cause immunosuppression (210, 214). Some studies have also found that NETosis can cause post-tumor thrombosis, further aggravating tumor damage e (211). Plasma DNA, neutrophil counts, and NET biomarkers have been suggested as diagnostic tools for assessing the propensity for thrombosis (215).

Many of the above findings suggest that research on tumor-induced NETosis should focus on targeting NETs that may benefit cancer patients. As research progresses, NETs have shifted from being initially considered a defense against serious infectious diseases to negatively impacting the body during cancer by promoting deadly processes such as thrombosis as well as systemic inflammation and cancer recurrence. With this in mind, NETs could provide excellent targets for future anticancer therapies.

## Conclusion

Cells can quickly disintegrate and die when exposed to extremely harsh environmental conditions, which is called accidental cell death (ACD). Minor exogenous or endogenous disturbances promote adaptive stress, thus restoring intracellular homeostasis. When the stress response fails to restore homeostasis, one or more signaling cascades are activated in the cell, contributing to the regulation of cell death.

There are many types of RCDSs with different mechanisms (**Table 3**). However, there is a very close relationship between many types of RCD. Although the application of RCD in cancer is mainly to promote the process of death, there is still a part of RCD that promotes the effect of cancer; hence, we cannot induce cell death for cancer treatment. As a bridge between different RCDSs, the caspase protein family, the Bcl-2 family, and the GSDM family are involved in most RCD processes and are representative markers of RCD. This also proves the connectivity between different RCDs from the side, which is also a breakthrough in the application of RCD in cancer treatment research. Currently, we have a better understanding of apoptosis, and we have learned about RCTs including necroptosis, pyroptosis, and ferroptosis, which remain to be understood, particularly the role of these processes in the development or treatment of cancer. The role of cell death in the tumor microenvironment is unique. From immunogenic cell death to immune checkpoint inhibition, various discoveries continue to remind us of the significance of cell death in cancer treatment. In addition to these common classic RCDs, many newly discovered types of RCDs are worth discussing. We believe that the widespread discovery of various non-caspase-dependent cell death means that our understanding of cell death is further advanced. The application of PARP inhibitors to improve the efficacy of chemotherapy is worth advocating. Various forms of death, including ferroptosis, play a double-role in cancer cells. Therefore, exploring the causes and mechanisms of their different outcomes in cancer patients is an issue that needs to be addressed.

Currently, we need to understand more about cell death, which will not only provide new ideas for current cancer treatment, but also provide more resources for cancer chemotherapeutic drugs, immunotherapy drugs, and targeted drugs.

## Author Contributions

GW conceived the idea of the manuscript. QL and XC undertook the initial research. XQ was involved in writing and plotting. QW reviewed and revised the manuscript. All authors contributed to the article and approved the submitted version.

## Funding

The PhD Start-up Fund of Liaoning Province from GW (2021-BS-209, Liaoning Province, 30000 CNY). Dalian Young Science and Technology Star from GW (2021RQ010 Dalian, Liaoning Province, 100000CNY).

## Conflict of Interest

The authors declare that the research was conducted in the absence of any commercial or financial relationships that could be construed as a potential conflict of interest.

## Publisher’s Note

All claims expressed in this article are solely those of the authors and do not necessarily represent those of their affiliated organizations, or those of the publisher, the editors and the reviewers. Any product that may be evaluated in this article, or claim that may be made by its manufacturer, is not guaranteed or endorsed by the publisher.
